# Way of Planning a Complex Interventional Treatment with Support of a 3-Dimensional Printed Heart Model in a Patient with Interrupted Aortic Arch Type A

**DOI:** 10.1007/s00246-022-03025-5

**Published:** 2022-10-28

**Authors:** Sabrina Martens, Dietmar Schranz, Hans-Gerd Kehl, Daniela Kiski

**Affiliations:** 1 Department of Cardiothoracic Surgery, University Hospital Muenster, Muenster, Germany; 2 Hessen Pediatric Heart Center Giessen & Frankfurt, Goethe University Frankfurt, Frankfurt, Germany; 3 Department of Pediatric Cardiology, University Hospital Muenster, Muenster, Germany

## Case Report

Complex heart defects occasionally pose special challenges for the physicians treating them. New techniques such as the use of 3D print files for individual planning are now being integrated into everyday clinical practice [1].

We would like to use the following case report to illustrate the increasing importance of this technology.

**Fig. 1:**
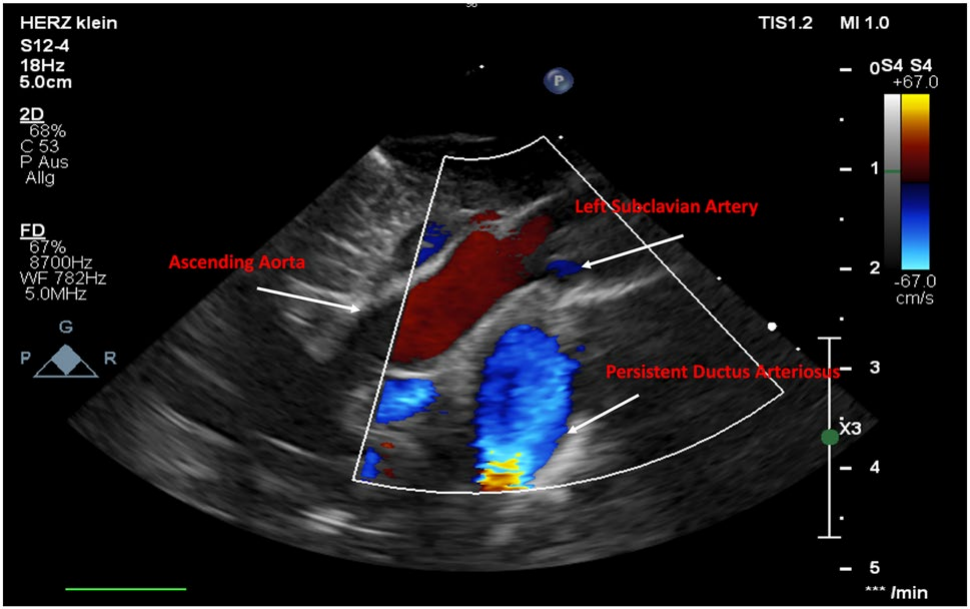
Echocardiography (suprasternal view) showing aortic arch interruption type A (IAA). Systemic circulation was secured by the Persistent ductus arteriosus (PDA)

After delivery with 38 + 6 weeks of gestation (birth weight of 2.35 kg) the prenatally assumed diagnosis of interrupted aortic arch type A was confirmed by transthoracic echocardiography (Fig. 1).

**Fig. 2:**
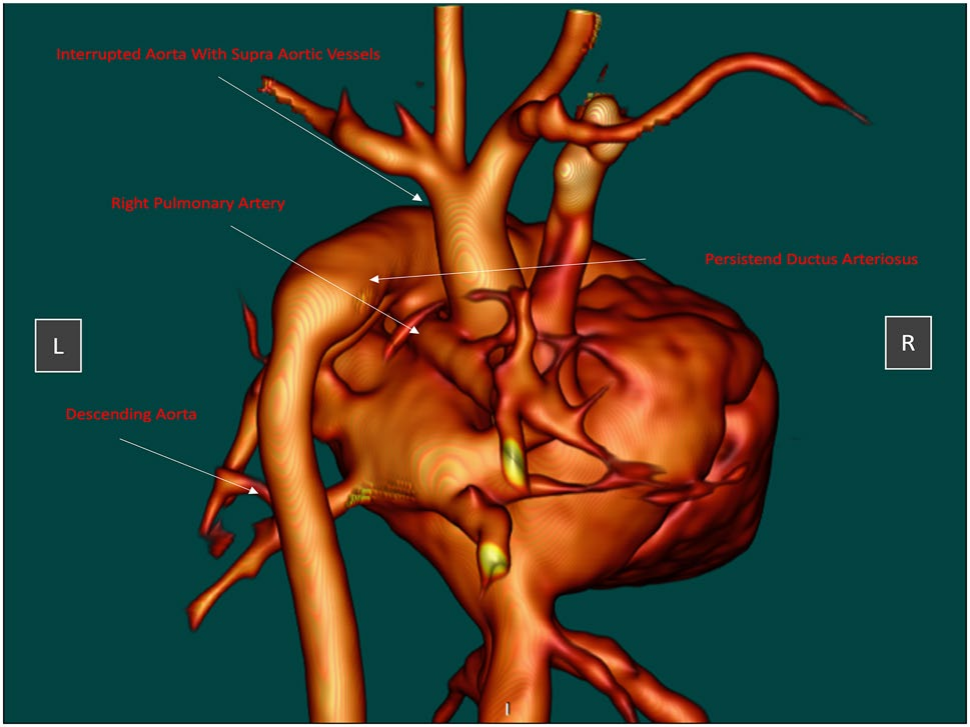
CT reconstruction

**Fig. 3:**
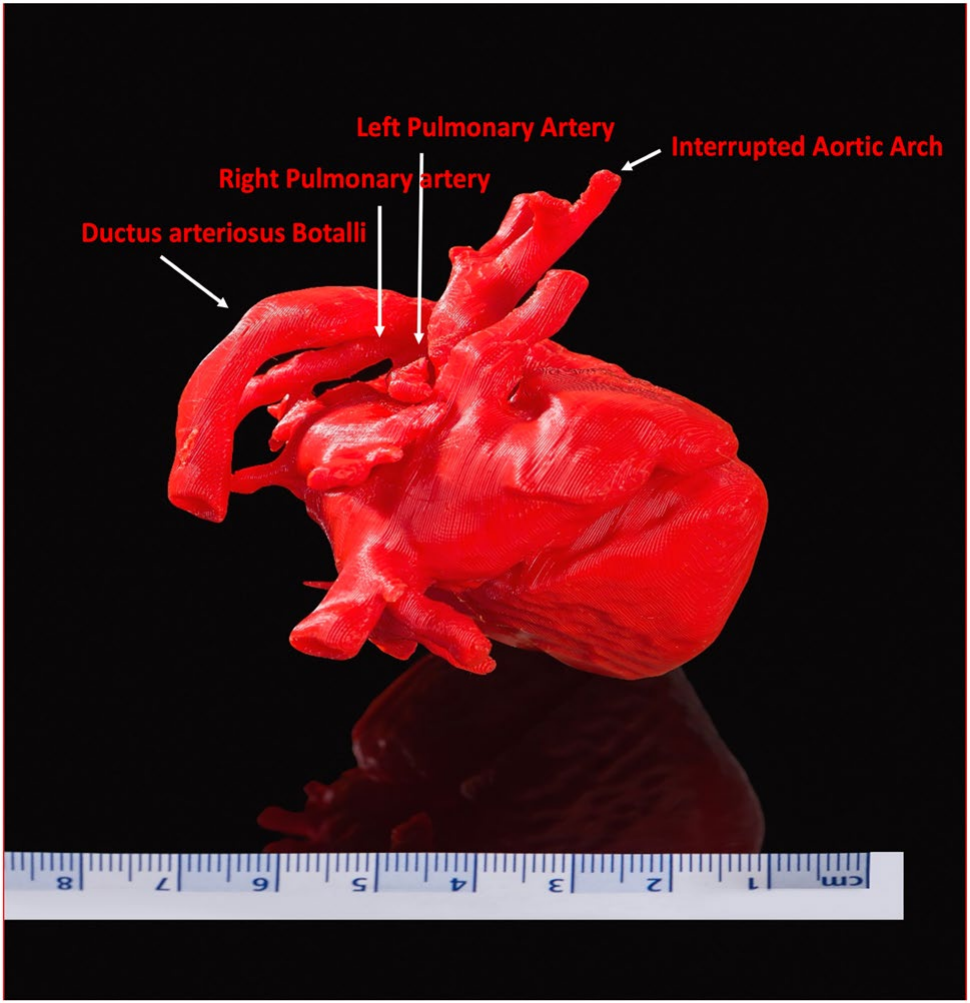
Printed 3-dimensional heart model (amplification 125%)

Additionally, a low-dose CT-scan was performed (Fig. 2). Based on these data we printed a 3D model to visualize exact anatomy. The data was modified with 3D Slicer Software^©^ and a 3D model was printed with a 3DGence Industry F340^©^ printer (Material ABS GP) (Fig. 3).

**Fig. 4:**
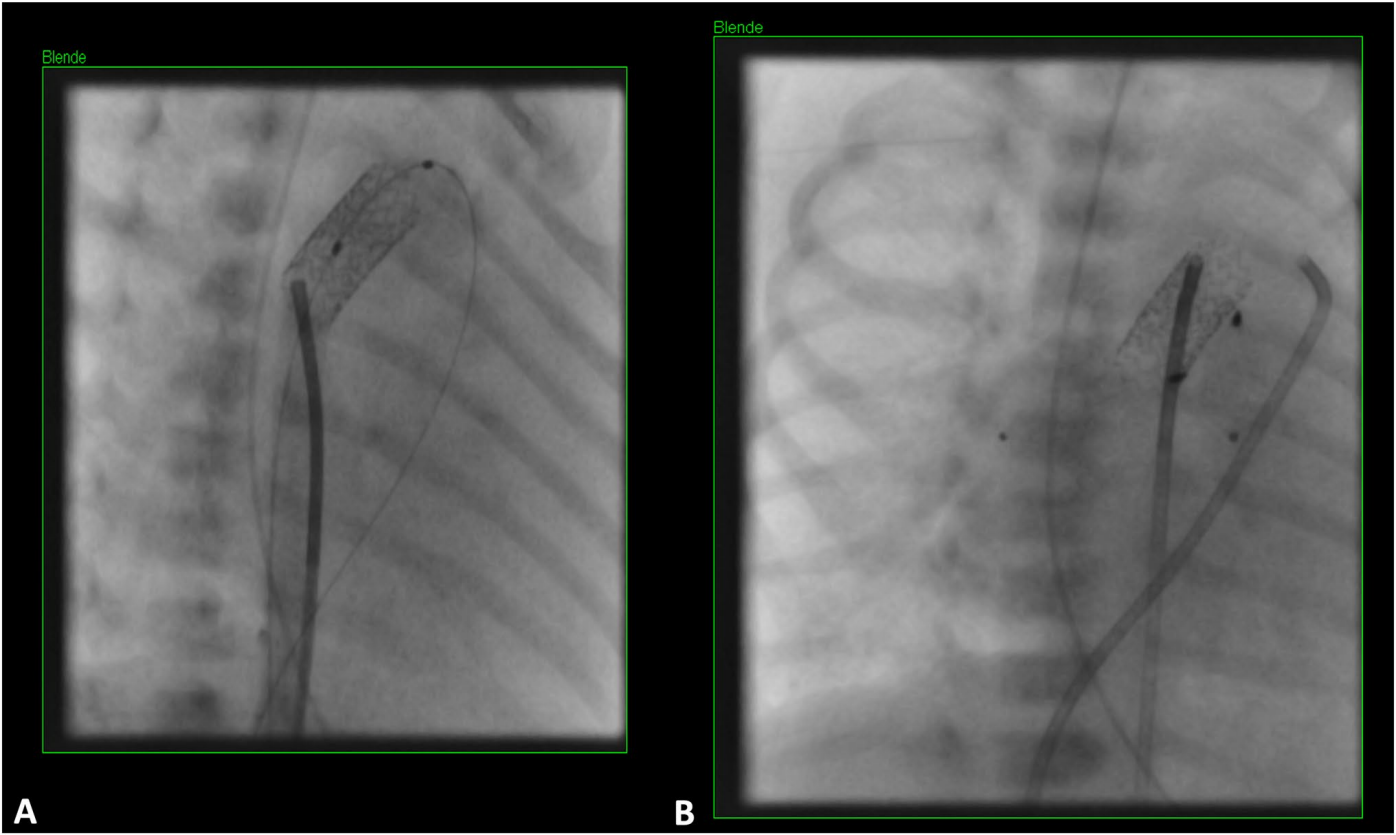
Catheterization with implantation of a modified 7 × 16 mm Formula Stents (10 atm) to keep the duct open and stepwise introduction of 7 mm modified Micro-Vascular Plugs (Medtronic MVP-7Q) in both pulmonary arteries to initialize intraluminal PA-Banding

An interdisciplinary team discussed different therapeutic options for the newborn child. Finally, interventional approach was preferred due to the low weight and young age of the patient. The child received a stent in the duct to ensure systemic circulation. Moreover, modified 7 mm Micro-Vascular Plugs (Medtronic MVP-7Q) were implanted into both pulmonary arteries to affect intraluminal PA-Banding (Fig. 4).

**Fig. 5:**
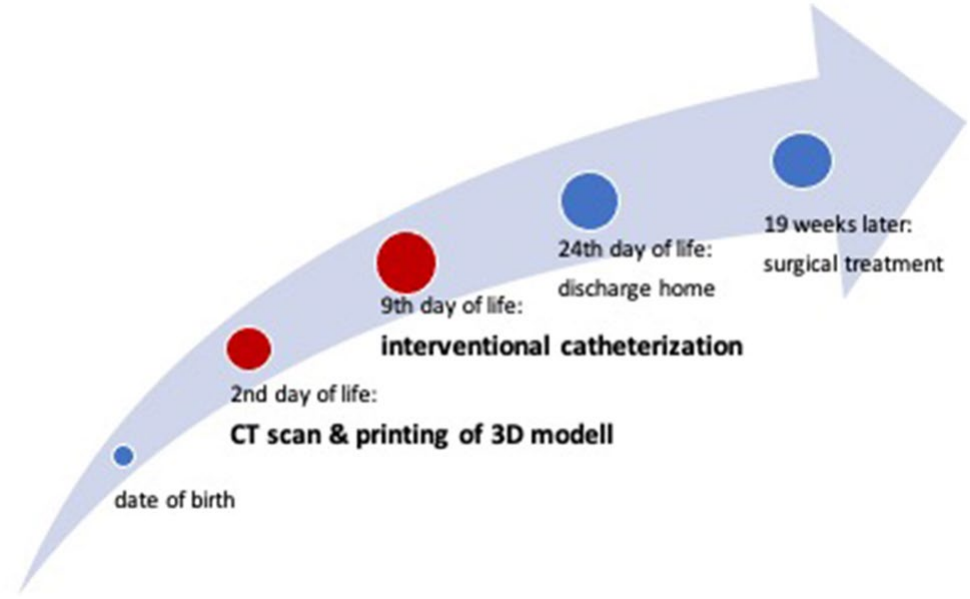
Timeline

In this way, the patient could be offered a safe therapeutic option. The child was able to thrive after intervention until surgical therapy months later (Fig. 5).

## Discussion

IAA Type A is a rare malformation. Most recently, different surgical and interventional approaches have been developed. In proposition to echocardiography and CT scan additional 3D models are well suitable supplements to visualize anatomical specialties which help to plan the procedure individually.

Due to the complexity of congenital heart defects and the small number of cases, there are only published case series and one study including 112 cases (96 for periprocedural planning and 16 for educational purposes [2]) on the use of 3D models in clinical practice.

Nevertheless, the use of 3D models for visualization of complex anatomic condition can be very helpful in the treatment of children with cardiac malformations. Patients do not only profit from more precise planning of any surgical or interventional procedure but also from a significant reduction of procedure times with sedation/narcosis, radiation dose and less periprocedural complications.

In the present case the knowledge of the gradual descent of the pulmonary arteries significantly shortened cardiac catheterization and associated anesthesia time. Thus, the modified 7 mm Micro-Vascular Plugs for intraluminal PA banding could be placed without complications. Stenting of the PDA also succeeded quickly. 19 weeks later corrective surgery was performed.

In summary, 3D models are very helpful for individual therapy planning and furthermore a good tool for education and training of medical staff [3, 4]. Even surgical strategy might be modified by the regular use of 3D models [5].

Another advantage of additional 3D printing might be the illustrative information and education of parents with regard to the illness of their child. Existing data files can be modified accordingly and different variants can be demonstrated [6]. Compliance and understanding might thus be increased.
